# The *Anopheles* community and the role of *Anopheles minimus* on malaria transmission on the China-Myanmar border

**DOI:** 10.1186/1756-3305-6-264

**Published:** 2013-09-13

**Authors:** Guo Yu, Guiyun Yan, Naixin Zhang, Daibin Zhong, Ying Wang, Zhengbo He, Zhentian Yan, Wenbo Fu, Feilong Yang, Bin Chen

**Affiliations:** 1 Institute of Entomology and Molecular Biology, College of Life Sciences, Chongqing Normal University, Chongqing, P.R. China; 2 Program in Public Health, College of Health Sciences, University of California, Irvine, California, USA; 3 Department of Pathogenic Biology, College of Medicine, Third Military Medical University, Chongqing, China

**Keywords:** *Anopheles* community, *An. minimus*, Blood meal, *Plasmodium vivax*, China-Myanmar border

## Abstract

**Background:**

Malaria around the China-Myanmar border is a serious health problem in the countries of South-East Asia. *An. minimus* is a principle malaria vector with a wide geographic distribution in this area. Malaria is endemic along the boundary between Yunnan province in China and the Kachin State of Myanmar where the local *Anopheles* community (species composition) and the malaria transmission vectors have never been clarified.

**Methods:**

Adult *Anopheles* specimens were collected using CDC light traps in four villages along the border of China and Myanmar from May 2012 to April 2013. Morphological and molecular identification of mosquito adults confirmed the species of *Anopheles.* Blood-meal identification using the female abdomens was conducted using multiplex PCR. For sporozoite detection in *An. minimus*, sets of 10 female salivary glands were pooled and identified with SSU rDNA using nested PCR. Monthly abundance of *An. minimus* populations during the year was documented. The diversity of *Anopheles* and the role of *An. minimus* on malaria transmission in this border area were analyzed.

**Results:**

4,833 adult mosquitoes in the genus *Anopheles* were collected and morphologically identified to species or species complex. The *Anopheles* community is comprised of 13 species, and 78.83% of our total specimens belonged to *An. minimus* s.l., followed by *An. maculatus* (5.55%) and the *An. culicifacies* complex (4.03%). The quantity of trapped *An. minimus* in the rainy season of malaria transmission was greater than during the non-malarial dry season, and a peak was found in May 2012. *An. minimus* fed on the blood of four animals: humans (79.8%), cattle (10.6%), pigs (5.8%) and dogs (3.8%). 1,500 females of *An. minimus* were pooled into 150 samples and tested for sporozoites: only 1 pooled sample was found to have sporozoites of *Plasmodium vivax*.

**Conclusion:**

*Anopheles* is abundant with *An. minimus* being the dominant species and having a high human blood index along the China-Myanmar border. The sporozoites in *An. minimus* were determined to be *Plasmodium vivax* with a 0.07-0.7% infection rate.

## Background

According to the *World Malaria Report 2012*, the estimated annual malaria incidence in the World and in South-East Asia for 2010 were 219 million and 32 million cases, resulting in 660,000 and 43,000 deaths, respectively [1]. Within SE Asia, the Greater Mekong Subregion (GMS) has been one of the most dangerous foci for malaria [2]. Nabang town established in 1996 is located in the western part of Yingjiang prefecture in the Yunnan province of China. It is a burgeoning treaty port with a total permanent resident population of 1,622 in 2010 and has a shared border with Lazan city in the Kachin state of Myanmar. A total of 372 malaria cases were confirmed by microscopic blood examination from this GMS border region between April and December in 2011. 60.2% of these cases originated from *Plasmodium vivax* (unpublished) and the other 39.8% came from *Plasmodium falciparum* (39.3%) and *P. ovale* (0.5%). In 2010, the annual malaria incidence rate in Yingjiang county of Yunnan province was 14.25 cases per 10000 people [3]. In the Kachin State of Myanmar, the rate was 21.7 cases/1000 people in 2006, and this area also had a high malaria-related mortality rate of 7.8 deaths/1000 people in 2005 [4].

Due to the outbreak of malaria, seasonal migrant workers, forest-related workers, pregnant women, <5 year-old children and miners have become high risk groups [5]. They continually cross the border to visit their relatives, trade goods or do other activities. They often contact malaria while travelling, resulting in an increase in imported malaria cases. For instance, one-third of malaria cases in China came from Yunnan province in 2005, and approximately a quarter of these were initially infected in Myanmar during trips to visit relatives and conduct business [6]. Up to now, most villagers live in wood dwellings with a thatched roof that mosquitoes can freely pass through. Many villagers have a low economic status and suffer from malnutrition. Moreover, there have been many regional wars between the Kachin Independence Organization and the central Union Government resulting in poverty and lack of development for the local population. Controlling malaria and providing effective treatment is problematic. Thus, malaria is a severe social and health problem in this border area between China and Myanmar.

Malaria has always been a serious public health problem, and the *An. minimus* complex and *An. dirus* s.l. have been reported as the malaria vectors in other regions of Myanmar, Yunnan and Hainan provinces of China, and are widespread all over the South and South-East Asia and southern China including the Ryukyu archipelago of Japan [7-11]. Previous studies around the border region have been carried out in 2007 [12,13]. Hardly any specific studies have focused on this area, and the local *Anopheles* community (species composition) and malaria vectors remained unknown.

The goals of the present research were to clarify the community of the local *Anopheles* community, survey the seasonality of predominant *Anopheles* species, investigate the preference of blood meal and the rate of infection with the *Plasmodium* parasite in *Anopheles* mosquitoes, and ultimately ascertain the main malaria vector and assess the significance of *An. minimus* s.l. for malaria transmission.

## Methods

### Study area

**Figure 1:**
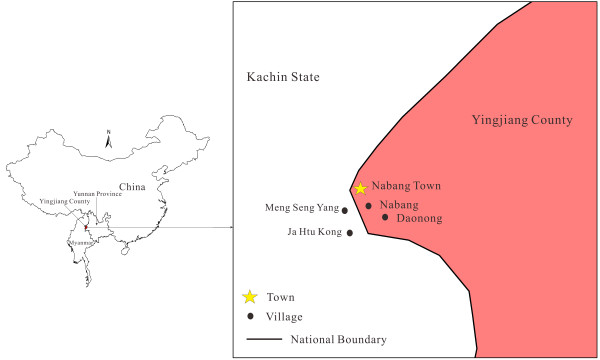
The map of sample collecting sites in the China-Myanmar border area.

This study was carried out from May 2012 to April 2013 in four villages in a China- Myanmar border area (Figure 1). Two villages each were located around Nabang town in Yingjiang county of China and in the Kachin region of Myanmar, respectively, namely Nabang (24˚72’63”N, 97˚57’27”E), Daonong (24˚67’05”N, 97˚58’49”E), and Mung Seng Yang (24˚72’77”N, 97˚55’68”E) and Ja Htu Kong (24˚70’63”N, 97˚56’60”E). The altitude of this mountainous district ranges between 180 and 1,200 m above sea level (mean: 240 m). This area belongs to a tropical rainy climate and is divided into a rainy season from May to October and a dry season from November to April. The average annual temperature is 22.7°C and the annual rainfall is 2,655 mm. Almost all investigated villages are located along the boundary river, the Lazan river. These villages are inhabited by people of the Jingpo nationality.

### Sample collection

CDC Mini light traps using incandescent lights (model 2836BQ, BioQuip Products, Inc. USA) were deployed for mosquito collection under roofs near the main entry door inside the house, which had no carbon-dioxide or other attractants for augmentation. During the study period, one village each in China and Myanmar was chosen to collect mosquitoes every three days, and 12 houses were selected randomly to trap mosquitoes in each village. Traps were set up between 19:00 and 07:00. Each morning, mosquitoes from the light traps were killed using ethyl acetate and subsequently counted. *Anopheles* mosquitoes were sorted and identified according to morphological characteristics using the keys compiled by Lu BL [14] and Dong XS [15]. Due to the existence of sibling species, the collected mosquitoes were morphologically identified by species complex rather than at species level. The cryptic species may be hidden based on morphological identification alone. After identifying the specimens, each mosquito was preserved in a plastic vial (1.5 ml) using absolute alcohol and stored at −20°C in preparation for DNA extraction, capped with silica gel, and subsequently taken to the laboratory.

### DNA extraction

All female adults of *An. minimus* were firstly identified by morphology before DNA extraction. The DNA was extracted from the abdomen of each mosquito for further species confirmation and blood-meal identification using multiplex PCR. Pooled samples comprising 10 thoraxes separated from female adult bodies were combined to extract the DNA for sporozoite identification [16,17]. DNA was isolated using a DNeasy Blood & Tissue Kit (Qiagen, Shanghai Co Ltd, China) with the extraction procedure following the tissue extraction protocol provided by the manufacturer’s instructions.

### Species identification by multiplex PCR

Specimens of *An. minimus* s.l. were distinguished and identified by multiplex PCR. 50 *An. minimus* specimens were selected from each village. The protocol was from Phuc *et al.*[18] and the primers were designed based on sequence variations in the second internal transcribed spacer (ITS2) of the ribosomal DNA (rDNA). The reaction mix consisted of 12.5 μl of 2 × Taq Master Mix, 0.5 μl of 5.8S forward, *An. minimus* and *An. harrisoni* primers (Table 1), 1 μl of template DNA and 10 μl of RNase free water, making a final reaction volume of 25 μl. PCR was performed under the conditions: 94°C for 5 min followed by 34 cycles of 94°C for 45 seconds, 51°C for 45 seconds, 72°C for 45 seconds and a final extension at 72°C for 10 min. *An. minimus* and *An. harrisoni* were characterized by 184 bp and 509 bp fragments. The 2 μl of PCR products were subjected to electrophoresis on a 1.5% agarose gel for *An. minimus* and *An. harrisoni*, and stained with ethidium bromide for visual detection using the Gel Imaging System (ChemiDoc™ XRS+, BIO-RAD, USA). If there was an expected result, the remaining sample of the amplified products was sequenced in both directions for confirmation.

**Table 1:** Primers used for multiplex PCR and nested PCR

**Species**	**Primer name**	**Sequences (5′ to 3′)**	**Amplification size (bp)**	**Sources**
Forward primer for *Anopheles*	ANF	ATCACTCGGCTCATGGATCG		Phuc *et al*. (2003)
*An. minimus*	MAR	GGGCGCCATGTAGTTAGAGTTG	184	
*An. harrisoni*	MCR	GGTTGCCCACTCAATACGGGTG	509	
Reverse primer for animal	UNR	GGTTGTCCTCCAATTCATGTTA		Kent *et al.* (2005)
Human	HUF	GGCTTACTTCTCTTCATTCTCTCCT	335	
Cow	COF	CATCGGCACAAATTTAGTCG	561	
Pig	PIF	CCTCGCAGCCGTACATCTC	453	
Dog	DOF	GGAATTGTACTATTATTCGCAACCAT	680	
*P. falciparum*	PFF	TTAAACTGGTTTGGGAAAACCAAATATATT	205	Snounou *etal.* (1993)
	PFR	ACACAATGAACTCAATCATGACTACCCGTC		
*P. vivax*	PVF	CGCTTCTAGCTTAATCCACATAACTGATAC	121	
	PVR	ACTTCCAAGCCGAAGCAAAGAAAGTCCTTA		

### Blood-meal identification by multiplex PCR

The identification of blood sources was carried out by using multiplex PCR as described by Kent *et al.*[19], with primers designed based on sequence differences in the *cytochrome b* of the mitochondrial DNA (mtDNA). Abdomens of blood-fed *An. minimus* were chosen with up to 50 from each site. When a site was too skimpy to select 50 abdomens, as many as possible blood-meals of *An. minimus* complex were selected. The volume system of the PCR reaction was similar to the species identification except for the primers used. Amplification was performed under the following conditions: 95°C for 5 min followed by 34 cycles of 95°C for 1 min, 55°C for 1 min, 72°C for 1 min and a final extension at 72°C for 10 min. A sample was considered positive when any fragments of four animals were detected. All PCR products were cloned, sequenced and blasted using NCBI BLAST.

### Sporozoite detection by nested PCR

The detection of sporozoites was adapted from the nested PCR described by Snounou *et al.*[20]. The following primer pairs were used: the plasmodium specific SSUrDNA primers rPLU5 (5′-CCTGTTGTTGCCTTAAACTTC-3′) and rPLU6 (5′-TTAAAATTGTTGCAGTTAAAACG-3′), which were used in an initial amplification reaction. The size of the DNA target is about 1,200 bp. These primers are genus specific and can amplify the target sequences from all four species of human malaria parasite, *Plasmodium falciparum*, *P. vivax*, *P. malariae* and *P. ovale*. The Plasmodium parasite species-specific SSUrDNA primers rFAL1, rFAL2, rVIV1 and rVIV2 were used for the specific detection of *P. falciparum* and *P. vivax* (Table 1). In the first round, DNA amplification followed the protocol: 10 μl of 2 × Taq Master Mix, 1 μl of rPLU5 and rPLU6 primers, 4 μl of template DNA, mixing in 20 μl final capacity with 4 μl of RNase free water. For the Nest 2, 2 μl of Nest 1 PCR products were used as the template for the second amplification where two pairs of species specific primers must be added. Amplification was performed under the following conditions: 2 minutes initial denaturation at 94°C, 30 cycles of 30 seconds at 94°C, 30 or 120 seconds at 58°C, and 120 seconds at 72°C, and a 5 minute final extension at 72°C. Nest 1 and 2 both obeyed this volume system and PCR procedure, with a small difference in the time at annealing temperature. Pooled samples were regarded as positive results if any fragment of 121 bp and 205 bp was obtained in the Nest 2.

## Results

### Species composition and *Anopheles minimus’s* seasonality

**Figure 2:**
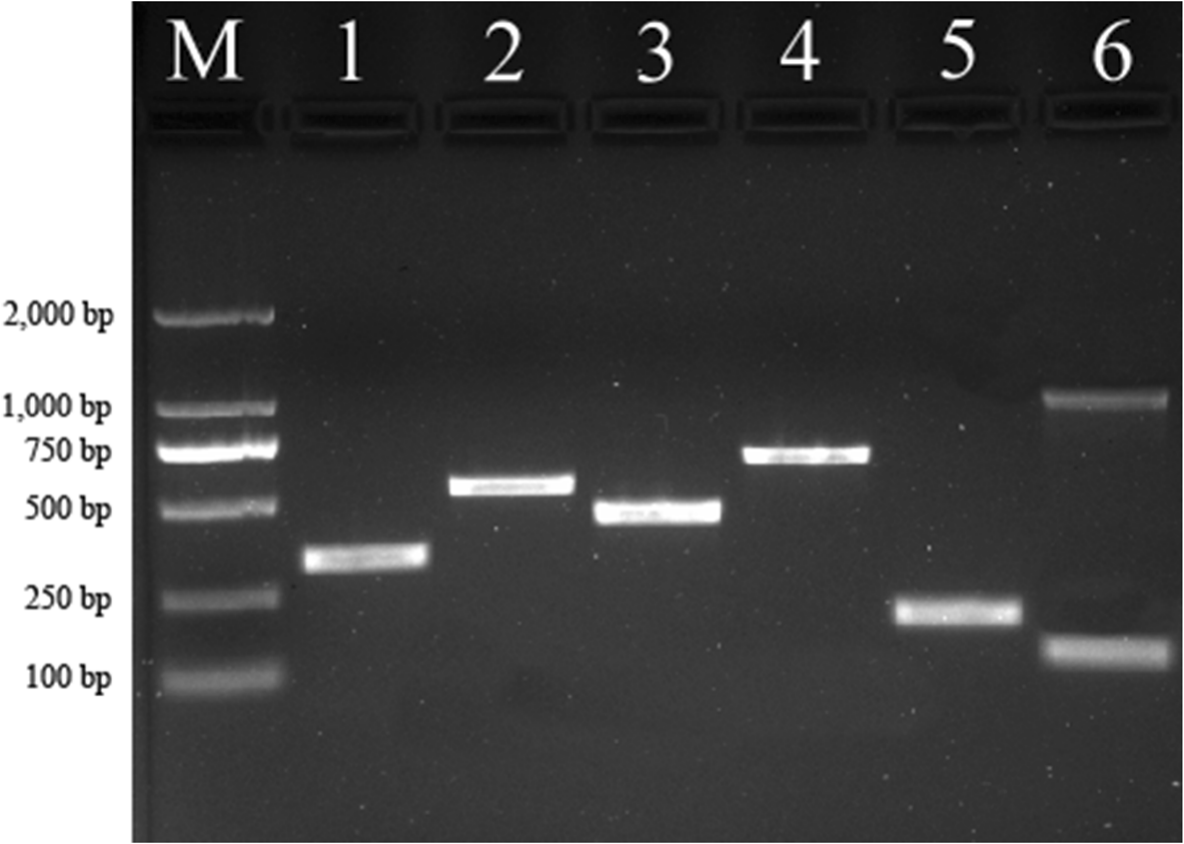
**PCR identification of *Anopheles minimus *s.l. species, blood-meal source and sporozoites.** The sizes of ethidium bromide-stained PCR products from multiplex PCR and nested PCR, examined by electrophoresis on agarose gel, served as the identification standard. Lane M: 2,000 bp ladder; lane 1: human; lane 2: cow; lane 3: pig; lane 4: dog; lane 5: *An. minimus*; lane 6: *P. vivax*.

During the year, a total of 4,833 adult *Anopheles* mosquitoes were collected using CDC light traps in households throughout the four villages. Among the collected mosquitoes, 13 species were identified based on morphological characteristics (Table 2). *An. minimus* s.l. was the predominant species representing 78.83% of the total number of trapped *Anopheles* mosquitoes, followed by *An. maculatus* (5.55%) and *An. culicifacies* complex (4.03%). Because of the small number of 37 *An. minimus* specimens collected in Daonong village throughout the year, a total of 187 morphologically-identified *An. minimus* s.l. were randomly selected for species confirmation by multiplex PCR followed by sequencing; all detected *An. minimus* specimens were confirmed as *An. minimus* based on the 184 bp fragment (Figure 2). Therefore, *An. minimus* was regarded as the only member of the complex in this region. This sequence was also blasted in NCBI for identification, and the result revealed a homology of 99% between the 184 bp fragment and the *An. minimus* sequence.

**Table 2:** **Community composition and individual percentage of *Anopheles *** **species in China-Myanmar border area**

**Species**	**Number of individuals**	**Individual percentage**	**Species**	**Number of individuals**	**Individual percentage**
*An. minimus*	3810	78.83	*An. peditaeniatus*	44	0.91
*An. maculatus*	268	5.55	*An. sinensis*	39	0.81
*An. culicifacies*	195	4.03	*An. kochi*	23	0.48
*An. jeyporiensis*	134	2.77	*An. tessellatus*	12	0.25
*An. vagus*	129	2.67	*An. messeae*	3	0.06
*An. splendidus*	104	2.15	*An. annularis*	2	0.04
*An. barbirostris*	70	1.45			

**Figure 3:**
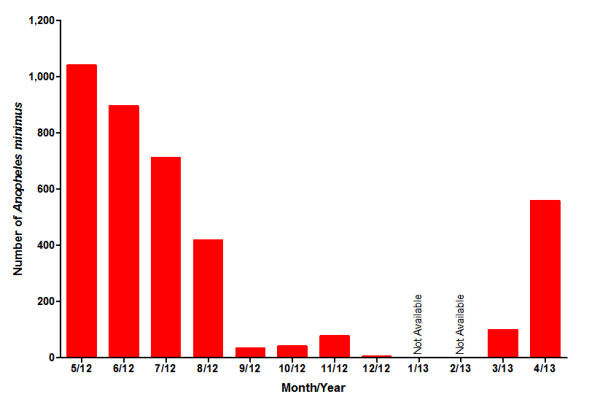
**Monthly abundance of *Anopheles minimus *** **in the China-Myanmar border area.**

The relative abundance of *An. minimus* s.l. varied throughout the year (Figure 3). Obviously the number of collected *An. minimus* in the rainy season of malaria transmission was greater than in the non-malarial dry season, and one high peak of abundance was found in May 2012 and the low peak period ranged from December 2012 to February 2013.

### Blood meal origin of *An. minimus*

Unfortunately, we failed to obtain any blood-meal in *An. minimus* from Daonong using the light traps, and only 4 blood-meal *An. minimus* s.l. specimens were collected in Nabang in the present study. Overall, 104 *An. minimus* specimens engorged with fresh blood from four villages were randomly selected to detect their blood meal source and identify the host species (Figure 2). The results show that 104 specimens confirmed as *An. minimus* gave positive reactions against human, cow, pig, dog or multiple antibodies (Table 3). Of all mosquitoes analysed, human beings were the primary host for blood meals accounting for 82.69% of the total number of detected specimens; this is significantly higher than other hosts. The proportion of cattle was 8.65%, pigs 4.81% and dogs 3.85%. In addition, 0.96% was discovered positive for blended blood meals of human and pig, and the mix of human and cow was also 0.96%. Therefore, the human blood index (HBI) of 82.69% for *An. minimus*, including mixed blood meals, was higher than the blood indices for cattle, pigs and dogs.

**Table 3:** **The numbers of total and blood-meal individuals from 4 collecting sites for *An. minimus***

**Collection sites**	**Total number**	**Number of blood-meal individuals**						
		Total (HBI*,%)	**Human (%)**	**Cow (%)**	**Pig (%)**	**Dog (%)**	**Human + Cow (%)**	**Human + Pig (%)**
Daonong	37	0 (0)	0 (0)	0 (0)	0 (0)	0 (0)	0 (0)	0 (0)
Nabang	275	4 (25)	1 (25)	2 (50)	0 (0)	1 (25)	0 (0)	0 (0)
Meng Seng Yang	922	50 (86)	41 (82)	4 (8)	2 (4)	1 (2)	1 (2)	1 (2)
Ja Htu Kong	2576	50 (84)	42 (84)	3 (6)	3 (6)	2 (4)	0 (0)	0 (0)
Total	3810	104 (82.69^§^)	84 (80.77)	9 (8.65)	5 (4.81)	4 (3.85)	1 (0.96)	1 (0.96)

*HBI – Human blood index. The number of Human + Cow and Human + Pig blood meals were added to the number of human blood meals when calculating the HBI. ^§^Overall HBI of *Anopheles minimus.*

### Sporozoite detection

A 121 bp fragment was amplified from one of the 150 pooled *An. minimus* samples (Figure 2). According to Snounou *et al*. [20] and Zakeri *et al*. [21], electrophoresis on a 1.5% agarose gel of the nested PCR products confirmed the existence of the *P. vivax* SSUrDNA fragment which was found to match with the previously reported result [20]. PCR analysis for detection of the *Plasmodium* genus and species determination established that the percentage of *P. vivax* infection was 0.07-0.7%.

## Discussion

In this study, we report our investigation of the *Anopheles* community and the role of *An. minimus* in malaria transmission in a region along the Sino-Myanmar border. According to our results, the *Anopheles* community in this district consists mostly of the primary vector *An. minimus* s.s. (occupying 78.83%) with another 12 *Anopheles* species accounting for 21.17%. A previous study reported the mosquito community near the China-Myanmar border in the western Yunnan, which included 12 *Anopheles* species with *An. sinensis* (accounting for 25.9%) being the most abundant species but *Culex tritaeniorhynchus* (67.9%) in this investigation [12]. However, the right China-Myanmar border area has a different environment and consequently different mosquito community composition, and the blood-meal and sporozoite of *Anopheles* mosquitoes have never been investigated before. Although several reports had confirmed that *An. sinensis* and *An. minimus* were among the major malaria vectors in China and Myanmar [22-25], there was no prior direct evidence for malaria transmission by the two species in this malaria prone area. The *An. dirus* complex has been reported in Myanmar [24] but it was not collected during this year-long study in this border region. Thus, *An. minimus* was regarded as the dominant endemic vector.

Because of the spread of the war between the KIO and the Central Union Government in this area between January and February 2013, work on mosquito collection was compelled to stop. Thus, complete data on seasonal variation during these two months was absent (Figure 3). Clearly *An. minimus* was seasonal in this border region where the warm and wet months of May-October were the peak season and the cold and dry months of November-April were the off season. The quantity of *An. minimus* in the rainy season far outstripped that in dry season. Meanwhile, *An. minimus’s* seasonality positively correlated with malaria occurrence in this district. Thus, effective preventive measures including free provision of bed nets and insecticide spray for each family must be available and implemented in a timely manner during the rainy period.

The research further indicates that *An. minimus* shows a variable behavior on blood meal. *An. minimus* s.l. comprised three sibling species, namely *An. minimus*, *An. harrisoni* Harbach & Manguin and *An. yaeyamaensis* Somboon & Harbach [26-28]. *An. minimus* and *An. harrisoni* have a broad distribution in much of Southeast Asia and Southern China [7]. However, most studies did not differentiate *An. minimus* and *An. harrisoni* on blood source detection. More investigations are required on *An. minimus* and *An. harrisoni* throughout an extensive geographical area where the *An. minimus* complex has existed. The *An. minimus* complex is either zoophilic or anthropophilic, depending on the local host availability. In this study, *An. minimus* fed on four animal’s (humans, cattle, pigs and dogs) distinguished by multiplex PCR. This population of *An. minimus* showed a greater bias for humans. The results of the high HBI for *An. minimus* is in line with previous research [11]. The high human preference of 82.69% suggests that malaria transmission tends to occur in this malaria epidemic region. The numbers for sites Daonong (37 individuals) and Nabang (275) are quite small, mainly because both are located in south-western Yunnan, where insecticide spray for each family was timely carried out and different economic crop planting resulted in adverse ecological environments for mosquitoes.

The identification of *Plasmodium* parasites in the salivary glands of *An. minimus* s.l. conclusively identified them as malaria vectors. Sporozoite infection rates were traditionally detected by dissection and inspection of the salivary glands of each mosquito under a light microscope. Molecular methods have been established in recent years, including the PCR method [29,30] aiming at amplifying specific DNA sequences for detection and CSP-ELISA detecting the circum sporozoite protein [11,31,32]. They have both advantages and disadvantages. In this study, a nested PCR method based on the amplification of the SSUrDNA of *P. vivax* sporozoites was selected to identify malaria vectors. This is a highly sensitive, conventional and effective approach. One of 150 pooled samples was confirmed positive in mosquito salivary glands by using this tool. It appears that *An. minimus* is the predominant malaria vector in this region.

In view of the findings of this study, *An. maculatus*, *An. jeyporiensis*, *An. culicifacis* complex and *An. sinensis*, the major malaria vectors reported in Thailand [33,34], India and Sri Lanka [35,36], and central China [37], respectively, are less abundant. That is not to say that these minor *Anopheles* populations do not have a potential vectorial role in the transmission of malaria in this boundary region. For instance, *An. maculatus* complex was infected by *Plasmodium* and also detected in Yunnan Province in south-west China [38]. They may be potential secondary malaria vectors and further research is required to understand the relationship between these Anopheline mosquitoes and malaria outbreaks. Malaria transmission will continue to be influenced by environmental changes, wars, dwelling conditions and human activities including visiting families and conducting making trade. Ultimately, this study provides baseline information for local evidence-based malaria control programmes, reveals valuable information necessary for the implementation of prospective control strategies, and will be useful to other regions or countries dealing with similar high burdens of malaria.

## Conclusion

The diversity of *Anopheles* mosquitoes on the China-Myanmar border area was abundant with 13 *Anopheles* species being identified based on morphological and/or molecular characteristics. *An. minimus* was the dominant species with a much higher human blood index compared to other animals in this area. Additionally, *An. minimus* was confirmed as the local malaria vector but with low sporozoite infection detected.

## Competing interests

The authors declare that they have no competing interests.

## Authors’ contributions

BC, GY and DZ conceived and designed the study, and BC also helped in performing data analysis and drafting the manuscript. Guo Yu designed the experiments, performed the study and drafted the manuscript. NZ, YW, ZH, ZY, WF and FY joined the specimens collecting and experiments. All authors read and approved the final version of the manuscript.
